# Compliance With Tobacco Control Policy and Visibility of Cigarette Retailers Around Educational Facilities in Riyadh, Saudi Arabia

**DOI:** 10.3389/fpubh.2022.713460

**Published:** 2022-05-30

**Authors:** Hala AlJishi, Dian Kusuma, Alaa AlQurashi, Ali AlFaiz, Abdulaziz AlSaad, Maha Aljishi

**Affiliations:** ^1^Research Services Administration, King Fahad Medical City, Riyadh, Saudi Arabia; ^2^Centre for Health Economics & Policy Innovation, Imperial College Business School, London, United Kingdom; ^3^Applied Clinical Research Administration, King Fahad Medical City, Riyadh, Saudi Arabia; ^4^College of Sciences, King Saud University, Riyadh, Saudi Arabia; ^5^Respiratory Administration, King Fahad Medical City, Riyadh, Saudi Arabia

**Keywords:** tobacco control, cigarette addiction, retailers, schools, Saudi Arabia (KSA)

## Abstract

**Background:**

In Saudi Arabia, cardiovascular diseases are among the top causes of death and disability, and smoking is one of the leading risk factors, particularly among males.

**Objective:**

Our study aims to evaluate the compliance with the anti-smoking law among cigarette retailers and examine the visibility of cigarette retailers around educational facilities in Riyadh city, Saudi Arabia.

**Methods:**

We conducted a mapping survey and geospatial analysis of cigarette retailers around educational facilities from February to March 2020 (before the COVID-19 restriction) in Al-Olaya municipality in Riyadh city as a pilot study. We found 249 retailers, of which 152 sold cigarettes. Data analyses in ArcMap 10.6 compared the visibility within 250 and 500 meters from educational facilities.

**Results:**

We found many retailers were not compliant with the tobacco control regulation: 57.1% of minimarkets sell cigarettes, 15.8% of cigarette retailers display the products openly, and 12.5% of cigarette retailers sold cigarettes by the stick. Moreover, 71% of the total cigarette retailers were within 500 m from schools, and 62% of all schools had at least one cigarette retailer within 500 m buffer (5-min walking or 2–3-min driving distance).

**Conclusion:**

There is non-compliance with the anti-smoking law among cigarette retailers and high visibility of cigarette retailers around educational facilities in Saudi Arabia. Monitoring is needed for the effective implementation of tobacco control policies.

## Introduction

Cardiovascular diseases (CVDs), including ischemic heart diseases and stroke, were the leading causes of deaths and disability globally in 2019 (1). CVDs contributed to 353 million DALY lost globally in 2016, of which 44% were attributed to smoking (2, 3). In Saudi Arabia, CVDs were among the top causes of deaths and disability, with tobacco as one of the leading risk factors, particularly among males (4). In 2013, the Saudi Health Interview Survey showed that smoking prevalence among 15+ years old males and females were 22.7 and 1.5%, respectively (5). Also, the mean age of smoking initiation was 19.1 years, with 8.9 % of ever smokers starting before 15 years (6). In 2018, another nationwide survey showed smoking prevalence among males and females aged 18+ years was 32.5 and 3.9%, respectively (7).

Saudi Arabia became a Party to the World Health Organization Framework Convention on Tobacco Control in August 2005. Aiming toward stricter tobacco control in the country, the national anti-smoking committee passed Royal Decree No 56, an anti-smoking law, in 2015 to combat tobacco use. It is the primary legislation governing smoke-free places, advertising, promotion and sponsorship; and packaging and labeling. Since 2016, smoking was banned in various settings, including mosques, ministries, government-owned factories, educational and health institutions, workplaces, and public transport. Moreover, the regulation bans selling cigarettes at smaller retailers such as minimarkets. It also prohibits tobacco product displays at the point of sales. Also, cigarettes must be sold in a packet, not by stick (8, 9).

While the progress toward comprehensive tobacco control regulation is promising in Saudi Arabia, evidence on the compliance is lacking. Also, evidence on the visibility of cigarette retailers (and product displays) around educational facilities is lacking. However, previous studies from other countries have shown that youth are highly receptive to tobacco advertising and restricting tobacco sales and advertisements near educational institutions may reduce students' tobacco use risk (10–12). Thus, our study aims to evaluate the compliance with the anti-smoking law, particularly among cigarette retailers, and to examine the visibility of cigarette retailers around educational facilities in Riyadh, Saudi Arabia.

## Methods

We conducted a mapping survey of retailers around educational facilities in Saudi Arabia, using Al-Olaya municipality in Riyadh city as a pilot study. There are two primary data, including retailers and educational facilities. First, we collected data on retailers from February to March 2020. We surveyed retailers including minimarkets (size of 24–99 square meters), supermarkets (100–499 square meters), and hypermarkets (500+ square meters). Before the COVID-19 pandemic, our trained data collectors surveyed the municipality area and recorded retailer geolocation, retailer type, cigarette product, product display, and loose cigarettes. Compliance was assessed by comparing our findings to the tobacco control regulation, which bans cigarette sales in minimarkets, open display of cigarette products, and selling cigarettes by the stick. For digital data collection, we used the KoboToolBox Android application, which is an open source, user-friendly and have been used in previous studies (13–15).

Second, educational facility data include a comprehensive list of government and private formal and informal educational facilities in the municipality. Formal facilities include kindergarten, elementary school, middle school, high school, and diploma/university; informal facilities include religious (e.g., Quran) schools. Data on schools, including addresses, were obtained from the Riyadh General Directorate of Education and the Ministry of Education. Because the KoboToolBox application was based on Google Maps, we used Google Sheets and geocoding add-ons to convert each facility address into geocodes (latitude and longitude). This technique has been well documented in the literature (16–18).

We conducted geospatial analyses in ArcMap 10.6 using Open Street Map as a base map. We employed several geospatial tools: (a) geoprocessing/buffer tool to generate buffers of 250 and 500 m around schools (14–16); (b) dissolved buffer to avoid duplication in calculation; and (c) spatial intersect and join tools to calculate the number of retailers around each school buffer or dissolved buffer. We used 250 and 500 m because the relatively smaller municipality area and almost all households drive in Riyadh city due to the very hot weather. We represented each school as a point on the map.

## Results

Table 1 shows the characteristics of schools and retailers in Al-Olaya municipality, Riyadh city. There is a total of 164 schools in our study (Table 1A), including 24.4% government schools and 75.6% private schools. A majority of the schools are all grades kindergarten to high schools (60.4%), others are elementary schools only (17.7%), kindergarten only (10.4%), middle school only (6.1%), and high school only (5.5%). In terms of retailers (Table 1B), there are 249 retailers in our analysis, including minimarkets (73.1%), supermarkets (24.9%), and hypermarkets (2.0%). The majority of retailers sell cigarettes (61%) while the rest do not (39.0%).

**Table 1:** Characteristics of sample in Al-Olaya municipality, Riyadh city.

		***n***	**%**
**(A) Schools**		164	
	Government	40	24.4%
	Private	124	75.6%
	All grades (kindergarten to high schools)	99	60.4%
	Kindergarten only	17	10.4%
	Elementary school only	29	17.7%
	Middle school only	10	6.1%
	High school only	9	5.5%
**(B) Retailers**		249	
	Hypermarket (500+ m^2^)	5	2.0%
	Supermarket (100–499 m^2^)	62	24.9%
	Minimarket (24–99 m^2^)	182	73.1%
	Sell cigarettes	152	61.0%
	Otherwise	97	39.0%
**(C) Cigarette retailers**		152	
	Hypermarket (500+ m^2^)	5	3.3%
	Supermarket (100–499 m^2^)	43	28.3%
	Minimarket (24–99 m^2^)	104	68.4%
	Number of cigarette products		
	1–6	7	4.6%
	7+	145	95.4%
	Product display at retailer		
	Yes	24	15.8%
	No	128	84.2%
	Sell by stick		
	Yes	19	12.5%
	No	133	87.5%

*m^2^, square meter. An example of cigarette products, Marlboro Gold is one product [Brand name = Marlboro, Product name = Gold)*.

Moreover, results also show non-compliance among cigarette retailers to the national tobacco control regulation (Table 1C). In terms of retailer type, the law bans cigarette sales in smaller retailers. However, of 152 cigarette retailers in our sample, we found 68.4% were minimarkets that are ban from selling. Among 182 total minimarkets in our study, 57.1% were found to sell cigarettes. Most of these cigarette retailers (95.4%) sell at least seven cigarette products. Also, we found 15.8% of cigarette retailers display the products openly, which is also banned under the regulation. Furthermore, we found 12.5% of cigarette retailers allow selling by stick, which is not permitted by the national policy.

**Figure 1:**
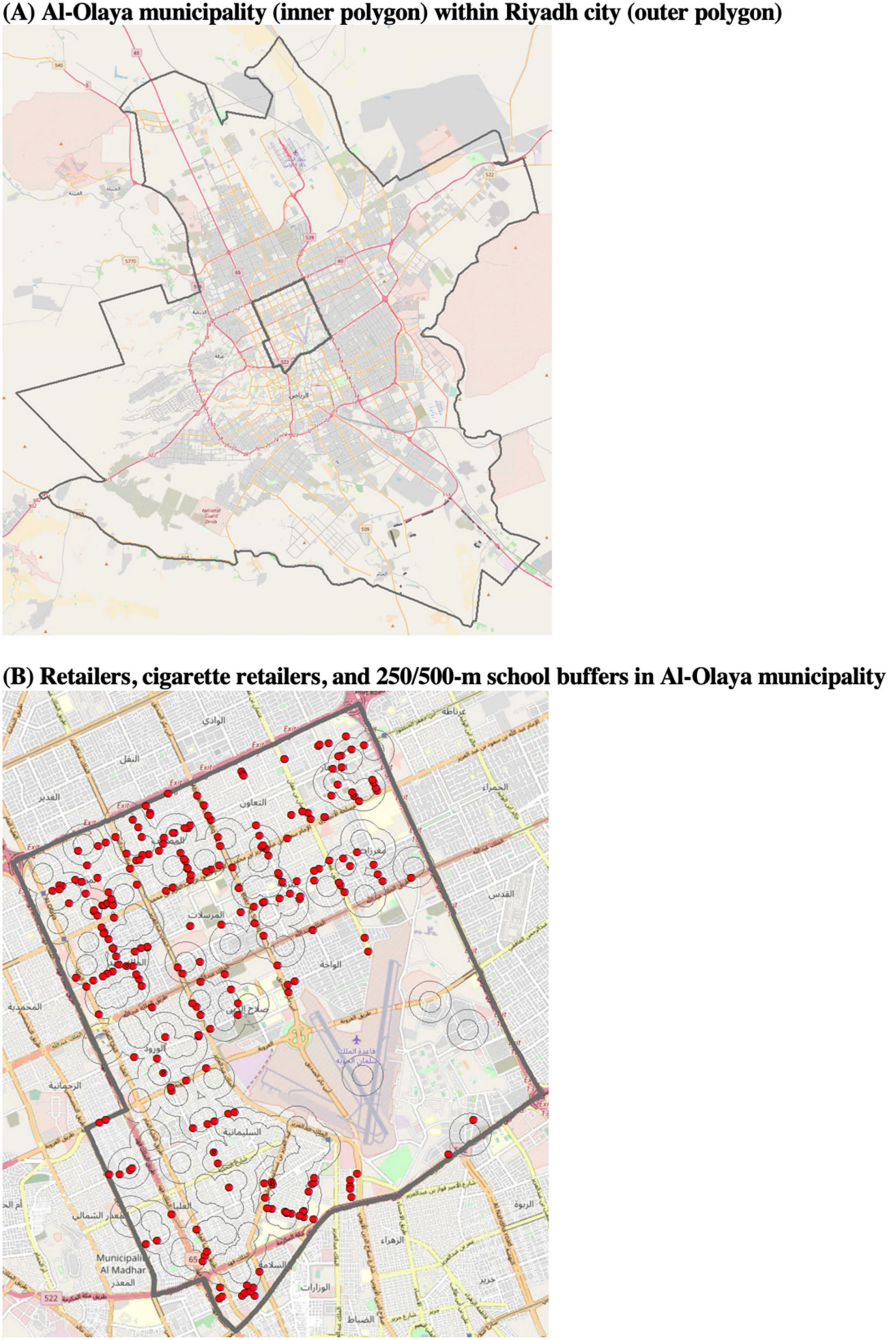
Visibility of cigarette retailers around schools in Riyadh city. **(A)** Al-Olaya municipality (inner polygon) within Riyadh city (outer polygon). **(B)** Retailers, cigarette retailers, and 250/500-m school buffers in Al-Olaya municipality. In **(B)**, red dots show retailers (including those selling cigarettes). The gray lines show 250-m and 500-m dissolved buffers around schools.

Figure 1 shows the maps of cigarette retailers and schools in Al-Olaya municipality, Riyadh City. In Figure 1A, the outer gray polygon shows the boundary of Riyadh city, and the inner gray polygon shows that of Al-Olaya municipality. In Figure 1B, red dots show all retailers, including those selling cigarettes, which we purposely do not reveal in a different color here. Gray lines show 500-m dissolved buffer around schools. Results show that schools and cigarette retailers are distributed in most of the municipality area, except for the south-east part of the study area, including an airbase. The mapping shows that many of the retailers are within the 500 m buffer from schools.

Table 2 shows the number of cigarette retailers within school buffers (Table 2A) and the number of schools with at least one cigarette retailer (Table 2B). Out of 152 cigarette retailers, 24% and 71% are within 250 and 500 m from schools, respectively. These figures vary by school ownership. Of the total cigarette retailers, 4 and 21% are within 250 m government and private school buffers; 31 and 54% are within 500 m government and private school buffers, respectively. Furthermore, out of 164 schools, 22 and 62% have at least one cigarette retailer within 250 and 500 m, respectively. These figures also vary by school ownership: 18% and 23% of government and private schools, respectively, have at least one cigarette retailer within 250 m buffer; and 65% and 60% of government and private schools, respectively, have at least one cigarette retailer within 500 m buffer. Distances of 250 m and 500 m correspond with 1–2 minutes and 2–3 minutes driving from schools, respectively.

**Table 2:** Cigarette retailers within buffer and schools with at least one cigarette retailers in Al-Olaya municipality, Riyadh city.

		** <250 m**		** <500 m**	
	**Total retailers**	**n**	**% of total**	**n**	**% of total**
**(A) Number of cigarette retailers within dissolved school buffer**					
All schools	152	36	24%	108	71%
Government schools	152	6	4%	47	31%
Private schools	152	32	21%	82	54%
		**<250 m**		**<500 m**	
	**Total schools**	**n**	**% of total**	**n**	**% of total**
**(B) Number of schools with at least one cigarette retailer within school buffer**					
All schools	164	36	22%	101	62%
Government schools	40	7	18%	26	65%
Private schools	124	29	23%	75	60%

*N, sample, m, meter. Calculations were conducted in ArcMap 6.10*.

## Discussion

We found that many retailers were not in compliance with the tobacco control regulation. Of the retailers, 57.1% of minimarkets sell cigarettes; 15.8% of cigarette retailers openly display the products; 12.5% of cigarette retailers sell cigarettes by the stick. Evidence from other countries is mixed. Studies found very high compliance rates of 97% immediately following the implementation of the ban of tobacco product display in Ireland (July 2009) and Norway (January 2010) (19, 20). However, other studies found relatively lower compliance in Indonesia (December 2017) (16). Furthermore, while countries like the United Kingdom and Sri Lanka have banned single cigarettes (21, 22), other countries like Indonesia and India have not (23, 24).

Moreover, we found that 71% of the total cigarette retailers in our analysis were within 500 m from schools. Also, we found that 62% of all schools had at least one cigarette retailer within a 500 m buffer (about 5–6-min walking or 2–3-min driving). This evidence shows high visibility and accessibility of cigarettes to young people at the educational facilities, which is a potential exposure to cigarette advertisement (e.g., through product display) and tobacco products in Riyadh, Saudi Arabia. Previous studies have found similar evidence in other settings. For example, a study among high school students in India showed that cigarette retailer density within 200–500 m of schools was associated with current tobacco use among students (11). Similarly, a study in Indonesia showed that youth at schools with a medium and high density of outdoor tobacco advertising was up to 2.16 times more likely to smoke, than those with low density (12).

For policy, our findings should provide preliminary evidence on the non-compliance with tobacco control regulation and the visibility of cigarette retailers near educational facilities in Saudi Arabia. There is a need to improve the compliance of the law for an effective policy in reducing tobacco control use, especially among young people in the country. However, our study has at least two limitations. First, because the study area is limited to a municipality within Riyadh city, the findings may not represent all regions. Second, this is a pilot study with a limited set of questions in the study instrument. Further research should expand the compliance assessment to include other aspects of the anti-smoking law and cover larger study areas, other cities, and rural areas to help improve its generalizability. Despite these limitations, we believe our findings have important policy implications for Saudi Arabia and beyond.

## Conclusion

There is non-compliance with the anti-smoking law among cigarette retailers and high visibility of cigarette retailers around educational facilities in Riyadh, Saudi Arabia. Further studies should cover larger areas of the country. Monitoring is needed for the effective implementation of tobacco control policies.

## Data Availability Statement

The raw data supporting the conclusions of this article will be made available by the authors, without undue reservation.

## Author Contributions

HA and DK conceived the study. HA, AAlQ, and AAlS conducted data collection. DK analyzed the data and drafted the manuscript. HJ, AAlQ, AAlS, AAlF, and MA provided inputs to the manuscript. All authors approved the final version.

## Conflict of Interest

The authors declare that the research was conducted in the absence of any commercial or financial relationships that could be construed as a potential conflict of interest.

## Publisher's Note

All claims expressed in this article are solely those of the authors and do not necessarily represent those of their affiliated organizations, or those of the publisher, the editors and the reviewers. Any product that may be evaluated in this article, or claim that may be made by its manufacturer, is not guaranteed or endorsed by the publisher.
